# Multiplex PCR Assay for Clade Typing of Salmonella enterica Serovar Enteritidis

**DOI:** 10.1128/spectrum.03182-22

**Published:** 2022-11-21

**Authors:** Sarah Gallichan, Blanca M. Perez-Sepulveda, Nicholas A. Feasey, Jay C. D. Hinton, Juno Thomas, Anthony Marius Smith

**Affiliations:** a Centre for Enteric Diseases, National Institute for Communicable Diseases (NICD), Johannesburg, South Africa; b Department of Clinical Microbiology and Infectious Diseases, School of Pathology, Faculty of Health Sciences, University of the Witwatersrand, Johannesburg, South Africa; c Department of Clinical Sciences, Liverpool School of Tropical Medicine, Liverpool, United Kingdom; d Malawi Liverpool Wellcome Research Programme, Kamuzu University of Health Sciences, Blantyre, Malawi; e Clinical Infection, Microbiology & Immunology, Institute of Infection, Veterinary & Ecological Sciences (IVES), University of Liverpool, Liverpool, United Kingdom; University of Sao Paulo

**Keywords:** nontyphoidal *Salmonella*, real-time PCR, phylogeny, molecular surveillance

## Abstract

Salmonella enterica serovar Enteritidis is one of the most commonly reported serovars of nontyphoidal Salmonella causing human disease and is responsible for both gastroenteritis and invasive nontyphoidal Salmonella (iNTS) disease worldwide. Whole-genome sequence (WGS) comparison of Salmonella Enteritidis isolates from across the world has identified three distinct clades, global epidemic, Central/East African, and West African, all of which have been implicated in epidemics: the global epidemic clade was linked to poultry-associated gastroenteritis, while the two African clades were related to iNTS disease. However, the distribution and epidemiology of these clades across Africa are poorly understood because identification of these clades currently requires whole-genome sequencing capacity. Here, we report a sensitive, time- and cost-effective real-time PCR assay capable of differentiating between the Salmonella Enteritidis clades to facilitate surveillance and to inform public health responses. The assay described here is limited to previously confirmed S. Enteritidis isolates.

**IMPORTANCE** Challenges in the diagnosis and treatment of invasive Salmonella Enteritidis bloodstream infections in sub-Saharan Africa are responsible for a case fatality rate of approximately 15%. It is important to identify distinct clades of S. Enteritidis in diagnostic laboratories in the African setting to determine the different health outcomes associated with particular outbreaks. Here, we describe the development of a high-quality molecular classification assay for clade typing of S. Enteritidis that is ideal for use in public health laboratories in resource-limited settings.

## INTRODUCTION

The key human pathogen Salmonella enterica has over 2,500 serovariants, determined by O and K surface antigens, and within individual serovars, there can be distinct pathotypes that currently require whole-genome sequencing (WGS) to identify. This diversity makes it challenging for surveillance systems to identify lineages of concern, and thus, the importance of specific variants is not communicated to public health authorities and policymakers. Sub-Saharan African (sSA) countries bear the greatest global burden of foodborne disease and are under pressure to increase production of protein-rich foods, often in the form of meat, but often have limited food, water, and environmental surveillance capacity (1).

The best available evidence suggests that animal source foods are the primary origin of foodborne pathogens in sSA (2). With global poultry production surpassing pork production in 2018, it is understandable that the poultry-associated nontyphoidal Salmonella (NTS) serovar Salmonella enterica serovar Enteritidis is the most-reported foodborne pathogen in sSA (3). Generally, S. Enteritidis infections are associated with outbreaks of gastroenteritis in Europe and the United States (4, 5). However, S. Enteritidis infections in sSA regions are commonly associated with severe, invasive bloodstream infections, known as invasive nontyphoidal Salmonella (iNTS) disease (6, 7).

The disproportionately high number of iNTS infections in sSA—approximately 79% of the global burden of iNTS (a 2017 estimate)—is closely associated with the high-risk populations in sSA (high numbers of advanced HIV infections, malaria cases, and young children with immature immune systems) (7). The high prevalence of immunosuppressed individuals in sSA has facilitated the emergence of iNTS as a major public health problem, with the two key serovars being S. Enteritidis and *S.* Typhimurium (8, 9) A 2016 study investigated the diversity of S. Enteritidis in sSA and, in addition to a globally prevalent poultry-associated lineage, identified two geographically distinct groups of S. Enteritidis strains circulating in sSA, namely, the West African and Central**/**Eastern African (East African) clades (8). The West and East African clades were quite distinct from the S. Enteritidis strains commonly associated with global gastroenteritis outbreaks, the global epidemic clade, raising the possibility of different ecological niche adaptation (8).

Despite the recognition of distinct S. Enteritidis clades and the severity of iNTS disease, the distribution and epidemiology of these clades across sSA remain poorly understood (10, 11). The lack of data pertaining to S. Enteritidis clades in sSA is, in part, due to the lack of a distinct molecular typing system for S. Enteritidis (12–14). The closely related *S*. Typhimurium has similarly unique clinical and epidemiological characteristics between its subtypes that can be clustered using multilocus sequence typing (MLST). Indeed, sequence type 313 (ST313) has been associated with epidemics of bloodstream infection, in contrast with the globally distributed ST19, which is mostly associated with gastroenteritis (15, 16). However, MLST fails to distinguish between S. Enteritidis variants, with the majority of isolates being assigned to ST11 (17). This becomes epidemiologically problematic when outbreaks of pathologically distinct S. Enteritidis clades are treated as a singular sequence type.

For public health officials and policymakers to both be aware of iNTS as a cause of severe febrile illness and institute policy to interrupt transmission and prevent iNTS, there needs to be the capacity to make the distinction between the gastroenteritis-associated global clade and the multidrug-resistant, invasive infection-associated East and West African clades (12, 18). Currently, the best way to distinguish between S. Enteritidis clades is through whole-genome sequencing, which is not widely available in sSA (19). Ideally, regional public health laboratories need access to robust, accurate, and cost-effective tests with a rapid turnaround time capable of differentiating between genetically similar isolates in order to facilitate appropriate epidemiological investigation of distinct pathovariants.

The real-time PCR assay is a commonly used method for the highly specific and sensitive classification of foodborne diseases and thus is widely available (20). When the real-time PCR assay is multiplexed, it has the advantage of enabling identification of multiple pathogens with a single assay (20). The scalability and rapid turnaround time of real-time PCR assays are also beneficial for use in diagnostic settings (20). The aim of the real-time PCR assay developed in this study is to classify S. Enteritidis isolates into clades in order to assist laboratories in typing S. Enteritidis strains; thus, we hope to aid in the surveillance of variants with an identical antigenic formula but which require different public health responses.

## RESULTS AND DISCUSSION

### Oligonucleotide design.

A gene presence/absence matrix produced by a pangenome analysis of 12 control panel isolates was used to identify unique gene target sequences that distinguished the clades associated with different geographical regions. These included the BTN76_08545 gene (protein family, NCBI protein accession number WP_023229131.1) for the African region and the SEN1975 gene for the global region (protein family, WP_001075993.1). Individual clades were recognized using the SEN1943 gene (protein family, WP_058658682.1) for the global epidemic clade and the *pemI* gene (protein family, WP_096198836.1) for the East African clade. To determine the sensitivity of the selected genes, a multilocus query based on the presence/absence of the genes BTN76_08545, SEN1975, SEN1943, and *pemI* in the whole-genome sequences of 500 S. Enteritidis isolates was performed using EnteroBase v. 1.1.3. Compared with the clade outcome predicted using the hierBAPS algorithm on the 500 S. Enteritidis whole-genome sequences (10), the multilocus classification was 90% effective in predicting the clade and 97% accurate in predicting the region of the S. Enteritidis isolate (see Table S1 in the supplemental material). The public health impact of an S. Enteritidis isolate being classified within the correct region and incorrect clade is minor, since the public health response for the region is the same as for individual clades. For example, S. Enteritidis isolates from the African region are associated with iNTS and multidrug resistance and therefore will require an appropriate public health response regardless of whether the isolate is classified further within the West or East African clade.

The four selected genes were then used to design primers and probes using the online PrimerQuest tool (Integrated DNA Technology; https://eu.idtdna.com/pages/tools/primerquest) (Table 1). The specificity of the designed primers and probes was tested on the whole-genome sequences of the 12 control panel isolates using the *in silico* PCR tool in CLC Genomics Workbench v. 11.0.1 (Qiagen, Hilden, Germany). The African cluster primer set amplified an 82-bp fragment of the BTN76_08545 gene for all six African isolates tested (isolates 10136/01, 0527/01, 8078/01, D7795, CP255, and 6396). The global region primer set amplified a 126-bp fragment of the SEN1975 gene for all six global region isolate sequences (isolates P125109, A1636, 1320, 791, 672246, and 672632). The East African clade primer set amplified a 101-bp fragment of the *pemI* gene from the East African clade isolate sequences (isolates D7795, CP255, and 6396). The global epidemic clade primer set amplified an 85-bp fragment of the SEN1943 gene from the global epidemic clade isolate sequences (isolates P125109, A1636, and 1320) (Table S2).

**TABLE 1:** Primer and probe sequences for the development of the Salmonella Enteritidis clade-typing real-time PCR assay

Target gene	Oligonucleotide name	Primer or probe sequence (5′ to 3′)	GenBank accession no.	Nucleotide position
BTN76_08545	African-F	TTGTATTGCGGTGGTACTCATA	CP018655.1	1645944–1646084
	African-R	AAACTCCGCACCTCCTAATC		
	African-FAM	56-FAM-TTACGCGGTTCGTTATGCGAGCTA-3IABkFQ		
SEN1975	Global-F	CTCGGTTTGGAGTTGTTGTTT	AM933172.1	2065272–2066153
	Global-R	CGTGCCAGATAGGCAGTATTA		
	Global-CY5	5CY5-TGACTGCTAGAGAGATGAGCGGTGA-3IABkFQ		
*pemI*	East-F	CTGTCGCTGGGTACAGATAATG	CP063703.1	99954–100054
	East-R	AACAGCTCAGCCAGTGAATAC		
	East-FAM	56-FAM-TGATAATGGCCGGCTGATTGTGGA-3IABkFQ		
SEN1943	Epidemic-F	TTTCTGTCAGCCAGTCCATTC	AM933172.1	2040288–2040905
	Epidemic-R	TACGTGGTTGCCTGATGTATTC		
	Epidemic-CY2	5CY5-TGCGTTACACGGACAACATCACCT-3IABkFQ		

### Validation of the real-time PCR assays.

The clade-typing real-time PCR assay strongly amplified (cycle threshold [*C_T_*], <30) the relevant target genes for all 12 control panel isolates listed in Table 2, allowing each isolate to be classified into the appropriate clade (Table 3). No weak positive (*C_T_* value, >30) or off-target amplification of the target genes was observed for the regional and clade real-time PCR assays (Table 3). Using a dilution series, the limit of detection was determined as the lowest DNA concentration resulting in a true positive (*C_T_*, <30). The limit of detection for these assays was determined to be 0.1 μM (Table 4).

**TABLE 2:** Salmonella Enteritidis strains used as the control panel in this study

Isolate name	NCTC no.^*a*^	Origin	Clade	Cluster	Reference(s)
P125109	13349	UK	Global epidemic	Global	21, 25
A1636	14674	Malawi	Global epidemic		8, 25
1320		Uganda	Global epidemic		This study
791		Uganda	Global outlier		This study
672246		South Africa	Global outlier		This study
672632		South Africa	Global outlier		This study
D7795	14676	Malawi	East African	African	8, 25
CP225	14675	DRC	East African		25
6396		Uganda	East African		This study
10136/01		Gambia	West African		22
0527/01		Gambia	West African		22
8078/01		Gambia	West African		22

a Strains with NCTC numbers are available from https://www.culturecollections.org.uk/.

**TABLE 3:** Average cycle threshold values from the clade-typing real-time PCR assays performed using the control panel isolates

Isolate name	Expected clade^*a*^	Real-time PCR *C_T_* value for target gene:^*b*^				Real-time PCR clade result
		BTN76_08545	SEN1975	*pemI*	SEN1943	
P125109	Global epidemic	−	19.31	−	17.72	Global epidemic
A1636	Global epidemic	−	19.26	−	18.12	Global epidemic
1320	Global epidemic	−	18.54	−	16.67	Global epidemic
791	Global outlier	−	19.9	−	−	Global outlier
672246	Global outlier	−	17.67	−	−	Global outlier
672632	Global outlier	−	18.23	−	−	Global outlier
D7795	East African	17.66	−	16.92	−	East African
CP225	East African	18.12	−	16.81	−	East African
6396	East African	17.05	−	16.6	−	East African
10136/01	West African	18.59	−	−	−	West African
0527/01	West African	18.74	−	−	−	West African
8078/01	West African	20.26	−	−	−	West African

a Derived from Feasey et al. (8) clade typing using whole-genome sequences.

b *C_T_* values under 30 indicate a positive result, and *C_T_* values over 30 indicate a negative result (−).

**TABLE 4:** Cycle threshold values from clade-typing real-time PCR assays performed with a DNA dilution series

Avg *C_T_* value ± SD at DNA concn (μM) of:^*a*^					Real-time PCR assay result
10	1	0.1	0.01	0.001	
23.33 ± 0.34	27.34 ± 0.08	28.89 ± 0.59	34.00 ± 0.54	35.98 ± 0.56	African region
21.95 ± 0.49	24.78 ± 0.74	28.96 ± 0.39	32.12 ± 0.46	35.15 ± 0.90	Global region
22.73 ± 0.48	26.08 ± 0.28	27.97 ± 0.42	32.62 ± 0.12	35.30 ± 0.09	East African clade
21.36 ± 0.26	23.99 ± 0.54	27.15 ± 0.49	30.05 ± 0.48	34.12 ± 0.49	Global epidemic clade

a Average of 3 replicates.

### Performance analysis of the real-time PCR assays.

To determine the assay efficiency, the regional and clade real-time PCR assays were performed using serial dilutions (10-fold) of the genomic DNA extracted from two control isolates (D7795 and A1636), and calibration curves were plotted to assess the linear range (assessment of how well the assay amplifies the target gene at various DNA concentrations [*R*^2^]) and the amplification efficiency (how well the assay amplifies the target gene region).

The regional real-time PCR assay that contained the African and global region primer and probe sets had linear ranges of 0.98 and 1.00, respectively (Table 5). The clade real-time PCR assay that contained the East African and global epidemic clade primer and probe sets had linear ranges of 0.99 for both (Table 5). Thus, the linear range for the clade-typing assay complied with the required *R*^2^ value of ≥0.98 (23), meaning that the primer and probes for the regional and clade real-time PCR assays efficiently amplified the target genes. The amplification efficiencies were calculated based on the slope of calibration curves. The theoretical maximum amplification efficiency is 1.00, which indicates that the amount of product doubles with each cycle (24). The regional and clade assays performed at average efficiencies of 1.00 and 1.04, respectively (Table 5).

**TABLE 5:** Efficiency of multiplex assays based on the average *C_T_* values and performance analysis of assays^*a*^

Primer or probe set target	Avg *C_T_* value ±SD at DNA concn (ng/μL) of:					*R*² (95% CI)^*b*^	Slope	Slope-derived efficiency
	10	1	0.1	0.01	0.001			
African cluster	23.33 ± 0.34	27.34 ± 0.08	28.89 ± 0.59	34.00 ± 0.54	35.93 ± 0.65	0.98 (0.95–1.00)	0.3	1
Global cluster	21.95 ± 0.49	25.55 ± 0.81	28.96 ± 0.39	32.12 ± 0.46	35.15 ± 0.90	0.99 (0.98–1.01)	0.3	1
East African clade	22.73 ± 0.48	26.08 ± 0.28	27.97 ± 0.42	32.62 ± 0.12	35.30 ± 0.09	0.99 (0.98–1.00)	0.31	1.04
Global clade	21.36 ± 0.26	23.99 ± 0.54	27.15 ± 0.49	30.05 ± 0.48	34.12 ± 0.49	0.99 (0.98–0.99)	0.31	1.04

a Performed with three technical replicates.

b *R*², determination coefficient; CI, confidence interval.

### Classification of clinical isolates.

All 618 S. Enteritidis isolates were successfully classified into clades using the multiplex real-time PCR assays reported here. The majority of S. Enteritidis isolates were classified within the outlier clade (377/618; 61.00%), with fewer classified within the global epidemic clade (240/618; 38.83%) and one isolate classified within the West African clade (1/618; 0.16%).

### Conclusion.

Here, we have described the development of a high-quality molecular classification assay for clade typing of S. Enteritidis that is ideal for use in public health laboratories, especially where WGS is not readily available. All primer and probe sets for the regional and clade assays ran at optimal efficiency within the multiplex assays. This novel multiplex PCR assay could be used to investigate whether certain clades of S. Enteritidis cause human disease of differing severity.

## MATERIALS AND METHODS

With respect to the phyletic structure of S. Enteritidis, we designed primers (regional and clade assays) to distinguish three clades and an outlier cluster in a single reaction. The purpose of the regional (African or global classification) and clade (global epidemic, global outlier, East African, or West African classification) assays is to further classify S. Enteritidis isolates to better understand the transmission and epidemiology of each S. Enteritidis clade. The regional and clade assays described here are limited to previously confirmed S. Enteritidis isolates.

### Control panel isolates.

The control panel consisted of 12 S. Enteritidis strains that were used as positive controls in the development of the multiplex real-time PCR assays. The 12 S. Enteritidis isolates were obtained as part of the 10,000 Salmonella Genomes project (25) and were selected based on the previously published S. Enteritidis global population structure predicted using the hierBAPS (hierarchical Bayesian analysis of population structure) algorithm (10). The control panel was assembled to represent the East African (*n* = 3), West African (*n* = 3), global epidemic (*n* = 3), and global outlier (*n* = 3) clades (Table 2). The clades were grouped into the global (global epidemic and global outlier) or African (East African and West African) regions (Table 2). All S. Enteritidis samples were stored at −70°C in 500 μL tryptic soy broth medium (1 L distilled water, 17 g casein, 5 g NaCl, 3 g soytone, 2.5 g dextrose, 2.5 g dipotassium phosphate, adjusted to pH 7.3).

### Genomic DNA extraction.

The control panel isolates were streaked onto 5% blood agar (Diagnostic Media Products, Johannesburg, South Africa) plates and incubated overnight in an IN 750 incubator (Memmert, Schwabach, Germany) at 37°C. Single colonies were resuspended in 400 μL of 10× TE buffer (800 mL distilled water, 2.92 g Tris, 15.76 g EDTA [pH 8]) in 2-mL Safe-Lock tubes (Eppendorf, Hamburg, Germany). The QIAamp DNA minikit (Qiagen, Hilden, Germany) was used to extract genomic DNA according to the instructions provided by the manufacturer. Final DNA concentrations were quantified fluorometrically using the Qubit 2.0 fluorometer (Thermo Fisher Scientific, CA, USA).

### Whole-genome sequencing.

The control panel isolates were sequenced and assembled as part of the 10,000 Salmonella Genomes Project using the LITE pipeline for library construction and the Illumina HiSeq 4000 system (Illumina, CA, USA) (25). The whole-genome sequences of all 12 S. Enteritidis isolates were annotated using Prokka v. 1.14.5 (26). The resulting annotated genomes were analyzed using ROARY v. 3.11.2 (27), producing a gene presence/absence matrix that compared the gene differences across the whole genome of each of the control panel isolates.

### Development of the multiplex real-time PCR assays.

**FIG 1:**
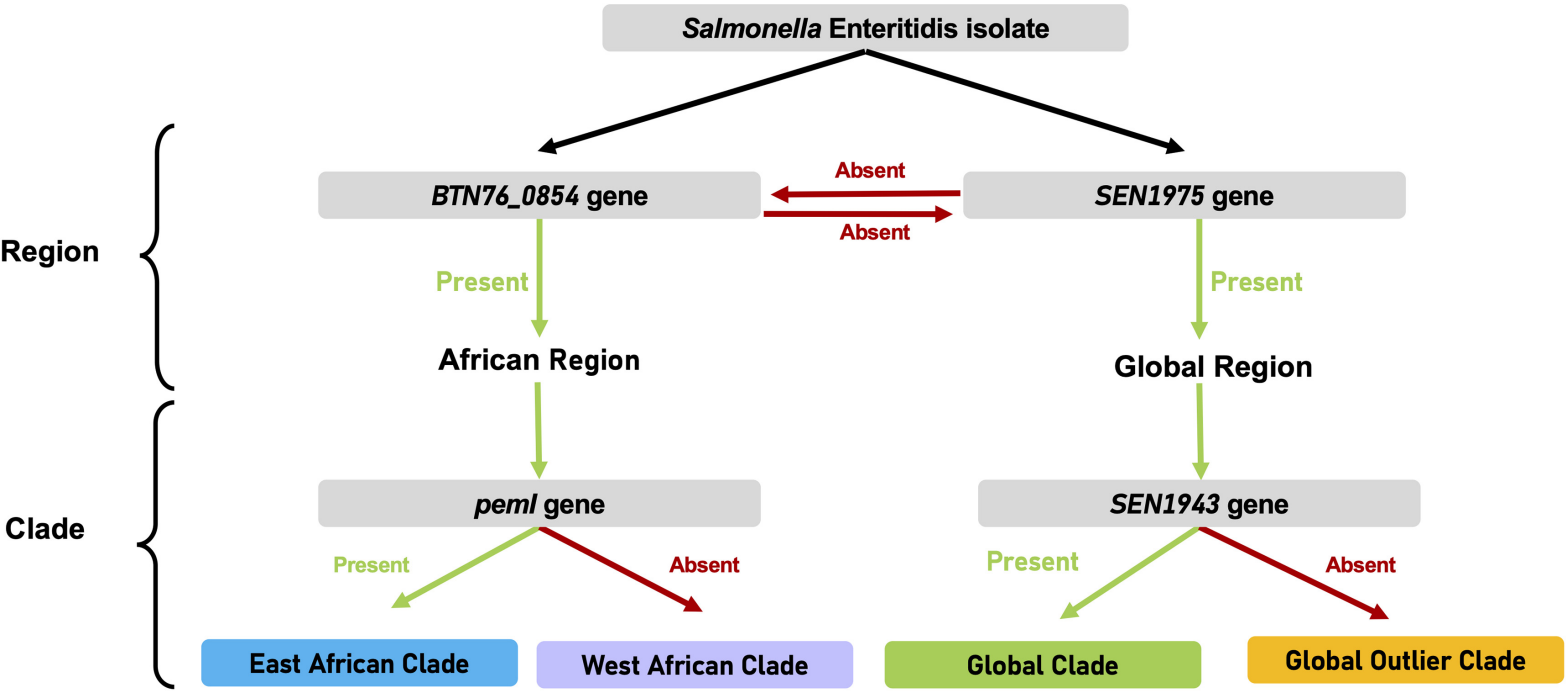
Workflow depicting the clade typing of a Salmonella Enteritidis isolate based on the presence or absence of genes targeted by the real-time PCR assay.

Target genes for the clade-typing real-time PCR assay were selected based on the presence/absence matrix (Fig. 1). To confirm the specificity of the selected genes, clade typing of 500 S. Enteritidis genomes was performed using EnteroBase v. 1.1.3. A workspace was created with the 500 S. Enteritidis genomes used in the published S. Enteritidis global population analysis (10) from whole-genome sequences obtained as part of the 10,000 Salmonella Genomes Project (25). The collection of 500 S. Enteritidis genomes consisted of clinical isolates from 45 countries and 6 continents, with representative isolates from the West African (*n* = 80), East African (*n* = 139), global epidemic (*n* = 195), and global outlier (*n* = 85) clades. A custom multilocus sequence typing analysis scheme using the target genes from the clade-typing real-time PCR assay was then used to type the S. Enteritidis genomes into clades. The clade results from this EnteroBase query were then compared with the S. Enteritidis global population structure predicted using the hierBAPS algorithm (8).

### Real-time PCR assay conditions.

All primers and probes were diluted to a concentration of 20 μM using nuclease-free water (Ambion, Thermo Fisher Scientific). Four master mixes for the two multiplex real-time PCR assays (regional and clade) were prepared as summarized in Table 6. A real-time PCR assay was set up using 25 μL TaqMan gene expression master mix (Thermo Fisher Scientific), 17.8 μL nuclease-free water (Ambion, Thermo Fisher Scientific), 3 μL of the relevant master mix (Table 6) (master mix 1 for the regional assay and master mix 2 for the clade assay), and 1.2 μL DNA template to each well of the MicroAmp Optical 96-well reaction plate (Applied Biosystems, Thermo Fisher Scientific). In each run, a negative control (1.2 μL nuclease-free water instead of DNA template) was added to the last well of the MicroAmp Optical 96-well reaction plate. The wells were then sealed with a MicroAmp Optical adhesive film (Applied Biosystems, Life Technologies, CA, USA) and centrifuged at 15,000 rpm for 1 min using an Allegra X-22R centrifuge (Beckman Coulter, CA, USA) to ensure that all reagents were concentrated at the bottom of the wells. The plate was then loaded into a 7500 real-time PCR system (Applied Biosystems, Life Technologies) and set up with the 7500 real-time PCR system v. 2.0 software (Applied Biosystems, Life Technologies). The reactions underwent PCR amplification as follows: 50°C for 2 min, followed by 95°C for 10 min and 40 cycles of 95°C for 15 s, 60°C for 30 s, and 72°C for 30 s.

**TABLE 6:** Constituents of the master mixes used in the regional and clade master mix real-time PCR

Primer or probe	Constituent			
	Regional master mix		Clade master mix	
	African	Global	Global epidemic	East African
Forward primer	African-F	Global-F	Epidemic-F	East-F
Reverse primer	African-R	Global-R	Epidemic-R	East-R
Probe	African-FAM	Global-CY5	Epidemic-CY2	East-FAM

### Multiplex RT-PCR assay performance.

To determine the efficiency of the multiplex real-time PCR assay, 10-fold serial dilutions of genomic DNA extracted from two control isolates (D7795 and A1636) were prepared. The DNA concentration of each dilution was quantified spectroscopically using a NanoDrop 1000 spectrophotometer (Thermo Fisher Scientific). A real-time PCR assay was then set up as described above using master mixes 1 and 2 for the regional assay and 3 and 4 for the clade assay. The DNA concentration yielding the highest *C_T_* value below 30 cycles was determined to be the limit of detection for that primer and probe set, in three technical replicates. The linear range (*R*^2^) was calculated for the *C_T_* values of the triplicate assays for each primer and probe set using the CORREL function in Microsoft Excel 2010. The slopes of calibration curves were used to calculate the amplification efficiency (PCR efficiency = 10^−1/slope^ − 1) (28).

### Classifying clinical isolates.

The multiplex real-time PCR assay was used to classify 618 clinical isolates, confirmed to be S. Enteritidis, into clades. The S. Enteritidis isolates were obtained from archived isolates submitted to the National Institute for Communicable Diseases of South Africa by four South African provinces (Gauteng, Mpumalanga, KwaZulu-Natal, and Western Cape) in the years 2012 and 2013.

### Ethical approval.

Ethical clearance for all laboratory-based surveillance and research (approved 12 November 2018) was obtained from the University of Witwatersrand, Johannesburg, South Africa (Wits protocol no. M140159), by the Centre for Enteric Diseases, National Institute for Communicable Diseases.
